# Linking gas production to microbial fuel cell output: a novel approach to assess soybean processing and selenium bioavailability

**DOI:** 10.1038/s41598-025-05608-7

**Published:** 2025-07-28

**Authors:** Vahid Vegari, Akbar Taghizadeh, Ali Hosseinkhani, Maghsoud Besharati, Kasim Sakran Abass, Maximilian Lackner

**Affiliations:** 1 Department of Animal Science, Faculty of Agriculture, University of Tabriz, Tabriz, Iran; 2 Department of Animal Science, Ahar Faculty of Agriculture and Natural Resources, University of Tabriz, Tabriz, Iran; 3 Department of Physiology, Biochemistry, and Pharmacology, College of Veterinary Medicine, University of Kirkuk, Kirkuk, 36001 Iraq; 4 Department of Industrial Engineering, University of Applied Sciences Technikum Wien, Hoechstaedtplatz 6, 1200 Vienna, Austria

**Keywords:** Soybean processing, Selenium supplementation, Microbial fuel cell, Rumen fermentation, Nutrient digestibility, Biotechnology, Microbiology, Systems biology

## Abstract

This study considered the effects of soybean processing methods (raw, roasted, microwaved) and selenium (Se) supplementation (nano-Se, sodium selenite) on in vitro rumen fermentation kinetics and microbial fuel cell (MFC) performance. Soybeans were thermally processed, and gas production (GP) and MFC voltage were measured over 96–120 h. Chemical analysis revealed microwave processing increased crude protein (39.20% vs. 37.35% raw) and reduced fiber content, enhancing digestibility. Gas production kinetics showed microwaved soybeans yielded the highest cumulative GP (312.75 mL/g DM at 96 h), surpassing roasted and raw treatments, likely due to structural modifications improving microbial accessibility. Nano-Se supplementation further amplified GP (320.04 mL/g DM at 96 h) and MFC voltage (3502.60 mV at 120 h), outperforming inorganic Se, attributed to enhanced microbial activity and antioxidant capacity. MFC voltage correlated strongly with GP (r = 0.95–0.99), validating MFCs as a dual-metric tool for assessing fermentation efficiency. Microwave processing generated the highest voltage (3241.30 mV), reflecting efficient electron transfer from disrupted fibrous structures. Nano-Se accelerated microbial kinetics, demonstrating superior bioavailability. Results highlight that thermal processing, particularly microwaving, optimizes nutrient utilization, while nano-Se enhances rumen microbial functions. The integration of GP and MFC metrics provides novel insights into feed degradability and microbial energetics, offering strategies to improve ruminant productivity and reduce environmental impacts. This study underscores the potential of combining advanced processing techniques and selenium supplementation to refine feed formulations and advance sustainable livestock practices.

## Introduction

The nutritional requirements of high-producing ruminants often exceed the microbial protein synthesized in the rumen, necessitating the inclusion of rumen-undegradable protein (RUP) sources that are digestible in the small intestine^1^. Soybean, a globally significant oilseed, is a low-cost, valuable source of protein (37–42%) and energy (18–22% fat), making it a critical component in dairy and ruminant diets^2^. However, raw soybeans contain anti-nutritional factors such as lipases and lipoxygenases, which can compromise fat quality and rumen microbial health^3^. Soybeans, depending on their processing method, can fulfill a portion of the degradable and non-degradable protein, energy, fat, and fiber requirements for dairy cattle, thereby serving as a valuable fat and protein supplement. They can be incorporated into ruminant diets in various forms, including raw, extruded, roasted, and microwaved. However, raw soybeans may contain certain enzymes, such as said lipases and lipoxidases, which can adversely affect the lipid content of the grain. Specifically, lipases can catalyze the release of free fatty acids from the oil present in the kernel, while lipoxidases can promote oxidative spoilage and the formation of peroxides, which may be detrimental to rumen microbes if ingested in excessive amounts. To mitigate the adverse effects associated with lipase and lipoxidase activity, it is advisable to utilize soybeans that have been subjected to heat treatment in the form of whole grains within the diet. Heated soybeans typically contain between 37 and 42 percent crude protein, 15 to 22 percent fat, and 12 percent moisture on a dry matter basis. Furthermore, the undegradable protein content of well-heated soybeans averages around 50 percent, which can be economically advantageous due to their energy, protein, and favorable digestibility and absorption characteristics^4^.

Studies highlight the potential of microwave-treated soybeans. This suggests enhanced microbial activity and nutrient accessibility^5^. Gas production kinetics, reflecting microbial fermentation efficiency, are influenced by substrate composition and processing methods. For instance, microwave processing may disrupt fibrous structures, accelerating microbial degradation^6^.

Concurrently, selenium (Se) supplementation has gained attention for its role in ruminant health and metabolism. Selenium, a trace mineral, is integral to antioxidant enzymes like glutathione peroxidase (GPX), which mitigates oxidative stress and supports immune function^7^. In ruminants, Se bioavailability is hampered by rumen microbial activity, which converts soluble Se into insoluble forms^8^. Organic Se (e.g., selenomethionine) and nano-Se exhibit higher absorption rates than inorganic selenite, improving tissue retention and antioxidant capacity^9,10^. For example, nano-Se increased GPX activity by 30% in sheep compared to selenite.

The interplay between soybean processing and Se supplementation may further optimize the rumen function. Selenium enhances microbial protein synthesis and volatile fatty acid (VFA) production, while processed soybeans provide fermentable substrates^11^. Microbial fuel cells (MFCs) offer a novel tool to study these interactions by quantifying electron transfer during fermentation, linking gas production to microbial electrochemical activity^12^. MFCs inoculated with rumen fluid can elucidate how Se and soybean processing alter microbial communities and energy harvest. For instance, higher gas production in microwave-treated soybeans may correlate with increased MFC voltage, indicating efficient electron transfer^13^.

By integrating gas production data with MFC analysis, this study provides a dual-metric approach to evaluate rumen fermentation efficiency. Findings could inform strategies to optimize feed processing and Se supplementation, enhancing ruminant productivity and reducing environmental methane emissions. This study investigates the impact of soybean processing (raw, roasted, microwaved) on MFC performance and in vitro gas production. The synergistic effects of Se forms (inorganic, organic, nano-Se) on fermentation kinetics and MFC output and correlations between gas production parameters and MFC metrics (voltage, power density).

## Materials and methods

The tested treatments include: (1) raw soybeans (2) microwaved soybeans (3) roasted soybeans (4) raw soybeans + nano selenium (5) microwaved soybeans + nano selenium (6) roasted soybeans + nano selenium (7) raw soybeans + sodium selenite (8) soybeans microwaved + sodium selenite (9) roasted soybeans + sodium selenite. The soybeans were purchased from a local feed store and were imported from Ukraine.

### Chemical analysis

The amount of dry matter, crude protein, NDF, ADF (neutral detergent fiber, acid detergent fiber) and crude fat was measured according to the methods suggested by AOAC^14^.

### Processing methods of tested soybeans

In the roasting method, the seeds were roasted for 5 min at temperature of 130°C in a roasting machine (Titan EF 6100 CF, 1400 Watt). A Panasonic 1200 W machine was used to microwave soybeans, and the seeds were microwaved for 2 min at 1200 W.

### Preparation of different sources of selenium

Different sources of selenium used in this study include: inorganic selenium as sodium selenite (Na_2_SeO_3_) (product of German Merck company with serial number 10102-18-8 and 99% purity) and nano selenium (product of German Serva company with 96% purity).

### Measuring the fermentability of feed by the gas production method

The methodology established by Fedorak and Hrudy^15^ was employed to quantify the gas production resulting from fermentation processes. Initially, 300 mg of both raw and processed seeds, which had been previously ground using a 2 mm sieve, were accurately weighed and placed into sterile bottles, with six replicates designated for each feed sample. Ruminal fluid was obtained from two sheep that had been slaughtered at a local abattoir and was promptly transported to the laboratory following filtration through a four-layer cloth and into a flask containing CO_2_. The ruminal fluid was then combined with a buffer solution prepared in accordance with McDougall’s method^16^ in a ratio of one part ruminal fluid to two parts buffer. To inhibit aerobic fermentation and maintain the temperature of the mixture, CO_2_ was introduced, and the solution was incubated at 39°C. Subsequently, 20 ml of the ruminal fluid and buffer mixture was added to each glass containing the sample. Following the injection of CO_2_ and the establishment of an anaerobic environment within the glass, the lids were securely sealed with rubber caps and aluminum foil before being placed in an incubator set at 120 rpm and 39°C. To account for gas production attributable to the ruminal fluid alone, three control bottles containing only 20 ml of ruminal fluid and buffer were prepared and incubated under the same conditions. The volume of gas produced was measured and recorded at intervals of 2, 4, 6, 8, 12, 16, 24, 36, 48, 72, and 96 h post-incubation.

## Microbial fuel cell (MFC)

**Fig. 1:**
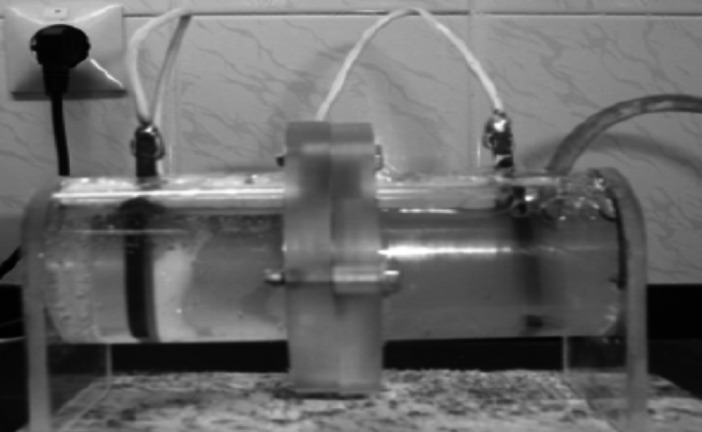
Dual-chambered microbial fuel cell setup.

Figure 1 illustrates the microbial fuel cell (MFC) configuration utilized in this study, which comprised three distinct setups. The experimental setup described herein comprises two plexiglass cylinders, each possessing a volume of approximately 125 cm^3^, with a 3 cm wide O-ring fitted aperture positioned between them to accommodate a 3.10 cm^2^ Flemion cation exchange membrane (Asahi Glass Co., Tokyo, Japan). The anode, designed in a rectangular shape, has a total geometrical area of 24.30 cm^2^ and is constructed from a commercial electrical-grade graphite plate. The spatial separation between the cathode and anode is measured at 8.50 cm. The composition of the catholyte solution includes a 60 mM biological phosphate buffer solution with a pH of 7.40, supplemented with MgSO₄ (0.90 g L^−1^; Merck) and NH₄Cl (1.00 g L^−1^; Merck, Germany). Ruminal fluid was collected following previously established protocols. Buffered rumen fluid, in conjunction with McDougall’s buffer (Merck; 100 mL), was combined with 1 g of each treatment and introduced into the anodic chamber. Following the inoculation of the bacterial culture into the anodic compartment, anaerobic conditions were achieved by sealing all openings around the anode with waterproof silicone sealant. The entire cell assembly was maintained at a temperature of 39 °C within a custom-built thermostatic chamber, a condition deemed essential for the growth and sustained activity of mixed culture bacteria. The potential between anode and cathode was measured and recorded at different hours^17^.

### Statistical analyses

The findings of the current study were evaluated utilizing a completely randomized statistical design, employing SAS^18^ statistical software for analysis. Duncan’s test was applied for the comparison of means at a significance level of 5%.

## Results and discussion

The approximate analysis results of the current experiment are given in Table 1. The table compares the chemical composition of raw, microwave-treated, and roasted soybean seeds on a dry matter (DM) basis. Thermal processing slightly increased the dry matter content, with raw soybeans at 94.6%, rising to 95.9% for microwave-treated and 96.1% for roasted soybeans. This suggests heat exposure reduces moisture content. Crude protein (CP) showed a notable increase in microwave-treated soybeans (39.20%) compared to raw ones (37.35%), likely due to protein concentration, while roasted soybeans had a slight dip to 38.07%, possibly from mild protein degradation. NDF and ADF content decreased with processing. NDF dropped from 25.10% in raw soybeans to 23.44% (microwave) and 22.70% (roasted), and ADF followed a similar trend, indicating improved digestibility as heat breaks down fibrous structures. Ether extract (EE), representing fat content, saw a minor decline in processed soybeans (25.29–25.67%) compared to raw ones (26.08%), potentially due to lipid oxidation. Ash content, reflecting minerals, increased slightly from 4.45% in raw soybeans to 4.60% (microwaved) and 4.72% (roasted), likely a concentration effect from reduced moisture. Microwave processing appears optimal for enhancing protein and fiber digestibility, while roasting offers similar fiber benefits with a small trade-off in protein. Fat and ash changes are minimal but may require monitoring for oxidative stability. Overall, thermal processing improves soybean nutrient concentration and digestibility, though statistical significance of these differences would need further validation.

**Table 1:** Effect of thermal processing type on the chemical composition of soybean seeds (%DM).

Feed Type	DM (%)	CP	NDF	ADF	EE	Ash
Raw Soybeans	94.6	37.35	25.10	15.80	26.08	4.45
Microwave-treated Soybeans	95.9	39.20	23.44	14.03	25.67	4.60
Roasted Soybeans	96.1	38.07	22.70	13.89	25.29	4.72

DM: Dry Matter, CP: Crude Protein, NDF: Neutral Detergent Fiber, ADF: Acid Detergent Fiber, EE: Ether Extract.

### In vitro gas production

#### Gas production for thermal treatments

The gas production from raw and processed soybeans was assessed at various incubation intervals, specifically at 2, 4, 6, 8, 12, 16, 24, 36, 48, 72, and 96 h, as detailed in Table 2. During the initial 24 h of incubation, the control treatment exhibited a significantly greater level of gas production compared to the other treatments. However, subsequent to the 24-h mark, the gas production levels in the microwaved and roasted treatments were found to be significantly higher than those observed in the control treatment. As seen in Table 2, there is a significant difference between the treatments in the early h of incubation. After 2 h, roasting and microwave processing caused a significant decrease in the amount of gas production, which continued until 24 h. Therefore, due to the high amount of fast-fermenting carbohydrates, more energy is provided for the growth and reproduction of microorganisms active in fermentation and gas production increases. Under controlled laboratory conditions, the incubation of feed with rumen fluid facilitates the conversion of carbohydrates into short-chain fatty acids, including propionic acid, acetic acid, butyric acid, valeric acid, and lactic acid, as well as the production of gases, predominantly CO_2_ and CH_4_. The Table 2 compares gas production (in mL/g DM) of raw, roasted, and microwaved soybean seeds over 96 h of incubation. Raw soybeans show significantly higher gas production (e.g., 150.30 mL/g at 8 h) compared to roasted (143.06 mL/g) and microwaved (138.52 mL/g) samples, likely due to untreated carbohydrates being more rapidly fermented by microbes. Gas production increases across all treatments, but microwaved soybeans begin to outperform roasted ones (e.g., 167.90 vs. 175.55 mL/g at 12 h). This suggests microwave processing may enhance digestibility over time. Microwaved soybeans consistently produce the most gas (peaking at 312.75 mL/g at 96 h), while roasted soybeans show moderate activity. Raw soybeans, despite early dominance, plateau earlier (262.38 mL/g at 96 h), possibly due to anti-nutritional factors degrading over time. Paya et al.^19^ reported that high gas production in soybean meal at different h of incubation can prove that the degradable protein in the rumen does not reduce the microbial activity in the rumen. Therefore, the high amount of gas production in raw soybeans can be attributed to not reducing microbial activity attributed to decomposable nitrogen in the rumen.

**Table 2:** In vitro gas production of raw and processed soybean seeds (mL/g DM).

Incubation time (h)	Raw Soybean	Roasted Soybean	Microwaved Soybean	SEM
2	19.85^c^	23.29^b^	26.11^a^	0.80
4	70.96^a^	68.84^b^	61.98^c^	1.20
6	109.43^a^	105.23^b^	100.31^c^	0.50
8	150.30^a^	143.06^b^	138.52^c^	0.40
12	184.75^a^	175.55^b^	167.90^c^	0.60
16	209.95^a^	205.85^b^	203.30^c^	0.50
24	229.56^c^	232.27^b^	235.58^a^	0.30
36	241.66^c^	251.07^b^	260.67^a^	8.50
48	250.42^c^	267.79^b^	283.96^a^	0.50
72	256.94^c^	276.89^b^	300.31^a^	0.50
96	262.38^c^	285.01^b^	312.75^a^	0.70

SEM: standard error means.

In general, gas production is the result of fermentation of carbohydrates to acetate, propionate and butyrate^20,21^. Gas production due to protein fermentation is relatively low compared to carbohydrate fermentation^22^. The contribution of fat is also partial in gas production^23,24^. It is important to highlight that the substantial volume of gas produced signifies elevated metabolic energy levels, as well as the presence of fermentable nitrogen and other essential nutrients required for microbial activity^23^. The formation of cross-links between amino acids and reducing sugars (Millard reaction) or between proteins (iso-peptide bonds)^25^ and the nature of proteins, can be responsible for the decrease in ruminal degradation of heat-treated protein^26,27^. The effect of heat on the amount of crude protein digestion depends on the moisture content of the grain, the temperature used and the duration of processing^28^. The alterations in the three-dimensional conformation of proteins finds in soybeans as a result of thermal treatment, along with the reduction in the digestibility of these proteins, may account for the diminished gas production observed in roasted and microwaved soybeans during this study. Parnian Khajehdizaj et al.^29^ reported that microwave irradiation increases the cumulative gas production of cereal grains, which is probably due to improved starch utilization through disruption of the protein matrix surrounding starch granules and increased susceptibility to enzymatic degradation, which is consistent with the data obtained in this experiment.

### Gas production for selenium containing treatments

Data related to gas production in different treatments at 2, 4, 6, 8, 12, 16, 24, 36, 48, 72 and 96 h of incubation are presented in Table 3. As can be seen from the results, the use of selenium supplements significantly increased the gas production potential during 96 h of incubation in different treatments (*P* < 0.05). The addition of selenium to livestock diet can affect rumen metabolism and function, especially microbial fermentation in it^30^. Nano selenium has been able to influence the average gas production in 24 h after incubation compared to the control treatment and lead to an increase in the volume of gas production (*P* < 0.05). Nano Selenium shows the highest gas production at all time points (e.g., 320.04 mL/g at 96 h), followed by inorganic Selenium and untreated soybeans. This suggests nano-selenium improves carbohydrate and protein digestibility, likely due to smaller particle size enhancing microbial activity. Inorganic selenium outperforms raw soybeans after 24 h (e.g., 231.06 vs. 211.28 mL/g at 24 h), indicating selenium’s role in mitigating anti-nutritional factors. Gas production increases logarithmically over 96 h, with the steepest rise between 2–24 h (e.g., raw soybeans: 18.37 → 211.28 mL/g). Nano Selenium exhibits the fastest fermentation kinetics, reaching 75.96 mL/g by 4 h (vs. 59.35 mL/g for raw soybeans).

**Table 3:** Gas production of soybean seeds with selenium additives (ml/g DM).

Incubation Time (hours)	Soybean	Soybean + Inorganic Selenium	Soybean + Nano Selenium	SEM
2	18.37^c^	22.57^b^	28.29^a^	0.80
4	59.35^c^	66.47^b^	75.96^a^	1.20
6	95.84^c^	103.16^b^	115.98^a^	0.50
8	130.56^c^	142.08^b^	158.52^a^	0.40
12	160.12^c^	174.09^b^	193.18^a^	0.60
16	187.99^c^	205.30^b^	225.81^a^	0.50
24	211.28^c^	231.06^b^	254.54^a^	0.30
36	227.47^c^	250.02^b^	276.44^a^	8.50
48	240.94^c^	265.91^b^	295.32^a^	0.50
72	249.02^c^	276.38^b^	308.74^a^	0.50
96	255.07^c^	284.75^b^	320.04^a^	0.70

The absence of similar letters in each row indicates a significant difference between treatments.

Wang et al.^11^ considered the increase in the concentration of volatile fatty acids produced in the rumen environment as evidence of the increase in ruminal fermentation of livestock with selenium supplementation. Hidiroglou et al.^31^ reported that the availability of selenium in the rumen can facilitate its use by rumen microbes and thereby improve the fermentation process in the rumen environment. Improving the fermentation pattern of the rumen environment leads to the improvement of gas production at different hours of incubation. The correlation of rumen fermentation with gas production was previously reported by Getachew et al.^32^. The volume of gas produced, which reflects the fermentation of feedstuffs to volatile fatty acids, can be an estimate of apparent digestibility^21^. The gas production potential was found to be greater in the treatments involving nano selenium and inorganic selenium, respectively, and this increase was statistically significant when compared to the control treatment (*p* < 0.05). However, no significant differences were observed among the treatments that utilized various forms of selenium. The rate of gas production in the control treatment was significantly higher than in other treatments. As can be seen from the results presented in Table 3, the use of selenium supplements significantly increased the gas production potential during 96 h of incubation in different treatments (*P* < 0.05). Naziroglu et al.^33^ reported that the addition of selenium and vitamin E to the diet increased the production of fatty acids and also increased the population of protozoa in the rumen environment. The fermentation of feed that results in a higher concentration of acetate is associated with an increase in gas production, in contrast to feed that promotes a greater production of propionate. This indicates that variations in the proportions of short-chain fatty acids correspond to alterations in gas production levels. Hidiroglou and Lessard^34^ and Wang et al.^11^ reported that dietary selenium intake increased the production of acetate, propionate and butyrate and total volatile fatty acids produced in the rumen. In general, gas production is the result of carbohydrate fermentation to acetate, propionate and butyrate^21^. Van Ryssen^35^ proved the existence of a positive correlation between the amount of gas produced and the level of selenium supplement fed to livestock and reported that the addition of selenium supplements at levels of 0.2 to 0.4 mg/kg DM in the livestock diet, can improve the fermentation pattern of the rumen environment, which is reflected in the promotion of gas production at different h of incubation. Also, this researcher reported that the production of short-chain fatty acids improved when taking selenium supplements at levels of 0.2 to 0.4 mg/kg DM.

### Relationship between volume of gas produced and MFC parameters of experimental treatments

The results obtained from microbial fuel cells are reported in Tables 4 and 5. All treatments show a logarithmic increase in voltage up to 120 h, with microwaved soybeans consistently producing the highest voltage (e.g., 3241.30 mV at 120 h), followed by roasted and raw soybeans. This aligns with prior findings that microwave processing improves nutrient accessibility for microbial activity. Raw soybeans initially generate the highest voltage (e.g., 268.00 mV at 2 h) but are overtaken by processed soybeans after 24 h, likely due to anti-nutritional factors in raw seeds hindering long-term microbial efficiency. Microwaved soybeans outperform others after 24 h (e.g., 2635.20 mV vs. raw’s 2611.30 mV), suggesting structural changes (e.g., reduced NDF/ADF) enhance electron transfer in microbial fuel cells. Roasted soybeans show intermediate performance, with smaller voltage gains compared to microwaved samples (e.g., 3143.60 mV vs. 3241.30 mV at 120 h). Nano selenium consistently generates the highest voltage (e.g., 3502.60 mV at 120 h), followed by inorganic selenium and untreated soybeans. This aligns with prior findings that nano-particles improve nutrient bioavailability, boosting microbial electron transfer. Inorganic Selenium shows intermediate performance (e.g., 3111.40 mV at 120 h), indicating selenium’s role in mitigating anti-nutritional factors. The voltage was found to increase logarithmically over 120 h, with the steepest rise between 2 and 24 h (e.g., soybean: 229.60 → 2448.70 mV). Nano selenium accelerates fermentation kinetics, reaching 2157.90 mV by 8 h (vs. 1899.50 mV for untreated soybeans).

**Table 4:** Microbial fuel cell voltage of raw and processed soybean seeds (millivolts/200 g DM).

Incubation time (hours)	Raw soybeans	Roasted soybeans	Microwaved soybeans	SEM
2	268.00 ^a^	258.70 ^b^	252.10 ^c^	1.359
4	944.10 ^a^	931.00 ^b^	919.70 ^c^	2.133
6	1522.20 ^a^	1503.60 ^b^	1483.20 ^c^	2.325
8	2046.42 ^a^	2018.60 ^b^	1988.70 ^c^	4.318
12	2385.60	2383.00	2380.50	5.440
24	2611.30 ^c^	2620.10 ^b^	2635.20 ^a^	6.062
36	2805.20 ^c^	2826.10 ^b^	2853.00 ^a^	8.611
48	2927.10 ^c^	2965.30 ^b^	3029.60 ^a^	10.826
72	3015.60 ^c^	3065.60 ^b^	3142.90 ^a^	21.950
96	3059.20 ^c^	3121.40 ^b^	3207.40 ^a^	25.490
120	3074.00 ^c^	3143.60 ^b^	3241.30 ^a^	28.041

**Table 5:** Microbial fuel cell voltage of soybean seeds with selenium additives (millivolts/ 200 g DM).

Incubation time (hours)	Soybeans	Soybeans + Selenium	Soybeans + Nano Selenium	SEM
2	229.60 ^c^	256.30 ^b^	292.70 ^a^	13.59
4	872.50 ^c^	923.70 ^b^	999.20 ^a^	21.33
6	1415.00 ^c^	1486.60 ^b^	1606.20 ^a^	23.25
8	1899.50 ^c^	1997.40 ^b^	2157.90 ^a^	43.18
12	2236.70 ^c^	2357.20 ^b^	2555.80 ^a^	54.40
24	2448.70 ^c^	2591.60 ^b^	2828.70 ^a^	30.62
36	2622.50 ^c^	2791.80 ^b^	3064.90 ^a^	86.11
48	2738.70 ^c^	2938.80 ^b^	3244.50 ^a^	88.26
72	2808.00 ^c^	3034.10 ^b^	3382.40 ^a^	59.50
96	2839.20 ^c^	3088.30 ^b^	3460.70 ^a^	54.90
120	2845.50 ^c^	3111.40 ^b^	3502.60 ^a^	80.41

Studies of microbial fuel cells have shown that the microbial composition of systems varies with substrate composition conditions and electrochemical performance^9,13^. However, extensive research is required to explore the impact of these effects on microbial populations. Ruminal fluid has been utilized as an inoculum in microbial fuel cells to identify biocatalysts that can generate electricity from cellulose^12^. Rismani Yazdi et al.^12^ conducted an experiment to identify electrogenic populations that can decompose cellulose and generate electrons. Their results showed that electricity can be produced using rumen bacteria from cellulose. Today, microbial fuel cells are rarely used in production and health studies of ruminants^12,36^. Microbial fuel cells are an advanced technology used to study microbial physiology in the form of electron (and proton) transport^6^. Microbial fuel cells offer a platform for the investigation of intricate microbial systems, including the rumen, while also presenting innovative methodologies for altering the interactions within these systems. A thorough comprehension of the diverse present within rumen microbial communities is likely to significantly enhance our understanding of nutrient digestion, production efficiency, metabolic disorders, and their environmental implications. Owing to their intrinsic capability for precise control of oxidation–reduction reactions, microbial fuel cells may assume a novel and significant role in the cultivation of rumen microorganisms. Studies of microbial fuel cells have shown that the microbial composition of the systems changes with respect to substrate composition conditions and electrochemical performance. Considering the effect of oil seeds on the oxidation and regeneration power of rumen bacteria by means of microbial fuel cells, the oxidation or regeneration power of bacteria can be evaluated according to the obtained voltage.

The correlation coefficients between the volume of produced gas and MFC are reported in Tables 6 and 7. The observed relationship between the amount of gas produced and the voltage obtained from the MFC indicates that the gas production is consistent with the data obtained from the oxidation–reduction of rumen bacteria, that is, by using fuel cells, the degradability of dry matter can be estimated with high accuracy.

**Table 6:** Correlation coefficients between thermal treatments and GP vs. MFC voltage.

Feed type	Correlation coefficient (GP vs. MFC)
Raw soybeans	0.9915
Microwaved soybeans	0.9518
Roasted soybeans	0.9726

**Table 7:** Correlation coefficients between selenium additives and GP vs. MFC voltage.

Feed type	Correlation coefficient (GP vs. MFC)
Soybeans	0.9821
Soybeans + Inorganic Se	0.9643
Soybeans + Nano Se	0.9917

Besharati et al.^37^ demonstrated that MFC traits exhibited the strongest positive correlations with various parameters, including in vitro gas production and chemical composition. The findings from the stepwise regression analysis indicated that the highest R-squared value was associated with non-fiber carbohydrates (NFC), which was incorporated into the model at the initial step. MFCs serve as a valuable instrument for investigating the physiological roles of microorganisms within intricate ecosystems. In contrast to gas production methods, MFCs generate electrons and protons from a substrate within an anode compartment. This process enables the potential quantification of feed components derived from the fermentation of substrates, thereby positioning MFCs as a viable technique for feed evaluation. Besharati and Taghizadeh^17^ demonstrated that MFC can serve as a novel and reliable assay for evaluating the nutritive value of ruminant feedstuffs, particularly whole cottonseed. Their study revealed strong correlations between in vitro gas production (GP) and MFC performance, with vitamin E supplementation enhancing both GP and MFC output (voltage, current, and power) by improving rumen bacterial activity. Conversely, monensin reduced GP but improved MFC efficiency, highlighting MFCs’ ability to capture metabolic shifts (e.g., propionate production) that GP methods may overlook. The findings underscore MFCs’ potential as an advanced tool for direct assessment of fermentable energy and microbial dynamics, offering advantages over traditional GP techniques. Further research is warranted to explore MFC applications in feed evaluation and rumen health.

## Conclusions

This study demonstrates a robust correlation between in vitro gas production (GP) and microbial fuel cell (MFC) performance, validating MFCs as a novel, dual-metric tool for evaluating rumen fermentation efficiency. Microwave processing of soybeans enhanced nutrient accessibility, yielding higher GP and MFC voltage compared to raw or roasted treatments, likely due to structural modifications improving microbial degradation and electron transfer. Nano-selenium supplementation further amplified both GP and MFC output, underscoring its role in enhancing microbial activity and antioxidant capacity. The strong correlations (r = 0.95–0.99) between GP and MFC metrics highlight MFCs’ ability to quantify real-time microbial electron flux, capturing metabolic shifts (e.g., propionate production) that traditional GP methods may overlook. By integrating fermentation kinetics with electrochemical data, MFCs provide a comprehensive assessment of feedstuff degradability and microbial energetics, offering insights into optimizing feed processing and selenium supplementation. This approach not only improves ruminant productivity but also aligns with sustainability goals by potentially reducing methane emissions. Future research should expand MFC applications to diverse feed substrates and microbial ecosystems, advancing precision in feed evaluation and rumen health management.

## Data Availability

Data is provided within the manuscript. The corresponding authors may be approached for more data.
